# Social Preferences for Orphan Drugs: A Discrete Choice Experiment Among the French General Population

**DOI:** 10.3389/fmed.2020.00323

**Published:** 2020-07-17

**Authors:** Mondher Toumi, Aurélie Millier, Olivier Cristeau, Katia Thokagevistk-Desroziers, Julie Dorey, Samuel Aballéa

**Affiliations:** ^1^Health Economics and Outcomes Research, Creativ-Ceutical, Paris, France; ^2^Research Unit EA 3279, Public Health Department, Aix-Marseille University, Marseille, France

**Keywords:** orphan drugs, rare disease, social value, equity, discrete choice experiment

## Abstract

**Objectives:** While several authors have suggested using a multi-criteria approach for orphan drug assessment and proposed lists of determinants of orphan drug value, studies on social preferences regarding these determinants remain limited. The current study aimed to identify preferences of the French general population regarding attributes characterizing the value of orphan drugs in a discrete choice experiment.

**Methods:** The list of attributes was formed based on a literature search and was refined through expert interviews, a focus group, and a pilot study. The final list included nine attributes: disease-associated disability, disease-associated mortality, number of patients, availability of alternative treatments, treatment impact on disease disability, treatment impact on mortality, treatment safety, uncertainty around therapeutic effect, and annual treatment cost per patient. Members of the General Public were presented with 12 choice sets containing two drug profiles described according to the attributes and an option to fund neither of these treatments. The questionnaire was disseminated online. A conditional logit model with random effects was used to estimate the weight of each attribute.

**Results:** A total of 958 persons participated in the study (48.7% male, mean age: 47.5 years). All attributes except for disease-associated disability had a statistically significant influence on the choices made by participants. The attribute with the highest weight was treatment impact on mortality (*p* < 0.0001), followed by uncertainty around therapeutic effect (*p* < 0.0001). The direction of results was generally consistent with intuition: patients preferred a drug with a larger impact on mortality, a larger impact on disability, with mild or no adverse events, with less uncertainty. Although patients appeared to prefer drugs with a lower budget impact, the relationship between patient preferences and costs was more complex.

**Conclusions:** Preferences of the general public between orphan drugs are mostly driven by the impact on mortality and the degree of certainty regarding the available evidence.

## Introduction

In 2004, NICE asked its Citizens Council to advise on whether or not the NHS should be prepared to pay premium prices for drugs to treat patients with very rare diseases (prevalence ≤1 per 10,000 persons) (1). The question was raised because the classic utilitarian approach in drug pricing which aims at maximizing the total health of the society conflicts with ethical principles of equity when it concerns orphan drugs (ODs). Indeed, the cost-effectiveness approach commonly used to inform decisions on drug coverage prioritizes common diseases over rare diseases because of lower per patient costs.

In NICE's survey, the majority of the Citizen Council members agreed that drugs for ultra-rare diseases should be treated differently than for common diseases. However, the support of the idea of paying premium prices for very rare diseases was conditional on certain circumstances. The rarity in itself was not judged sufficient to justify higher prices, and other criteria, such as the degree of severity of the disease, health gain (rather than just stabilization), and life-threatening nature of the disease, were cited as the most important for the decision. These findings represent a very strong affirmation of the complexity of ODs value which is defined by multiple parameters.

The inclusion of multiple criteria in the decision on ODs coverage was supported by many authors. A systematic literature search on elements of ODs value conducted by Pauldren et al. identified 19 candidate decision factors cited in the literature (2). Many authors discussed potential determinants of the social value of ODs and proposed alternative decision frameworks which considered multiple elements (2–6). Most of them included disease severity, availability of alternative options, drug efficacy, and innovation profile.

Despite the extensive discussion around the complex nature of ODs value, little is known about public preferences regarding ODs reimbursement. Few studies aimed at analyzing the social value of ODs and the willingness of the society to provide these treatments to patients (1, 7, 8). These few studies demonstrated contradictory results and revealed the complexity of the research question. The inconsistency of the findings lied in expressing the will for equity in treatment access, but refusing to prioritize rare diseases based on low prevalence or to value the rarity in and of itself. Drummond and Towse explained these contradictions by differences in understanding the notion of equity (9). They distinguished two types of equity: horizontal equity (equal treatment of equals) and vertical equity (unequal treatment of unequals) which might explain these seemingly contradictory findings. The authors emphasized the importance of explaining to participants the consequences of their choices and recommended using robust techniques for preference elicitation, such as DCE or conjoint analysis.

DCE is a preference elicitation technique which allows for estimating the impact of multiple criteria (attributes) on the final decision (10). DCE evaluates preferences of an individual by analyzing choices he/she makes while trading-off between a series of choice alternatives described by a number of predefined attributes. DCE mimics real choice behavior which is natural and easy to understand for participants, and it is less prone to bias than direct rating for assigning weights to attributes (11).

The objective of the present study was to identify drug and disease characteristics that are valued the most by the society and to estimate their relative weights. The study was designed as a DCE and conducted in the French general population.

## Methods

A DCE requires trading-off between a number of alternatives. The alternatives are described based on a set of predefined attributes. Each participant is presented with several choice situations comparing different alternatives (two or more alternatives per choice situation). The characteristics of the compared alternatives in each choice situation are defined in an experimental design. Different techniques exist to construct the experimental design with a common goal to maximize the information that can be obtained from the participants' choices. After the data collection phase, estimates of the mean weight of each attribute in the decision are obtained through a statistical analysis. The general study methodology followed the key stages of the development of a DCE, as suggested by the ISPOR Good Research Practices Task Force report on constructing experimental design for DCE (10). The selection of attributes and their levels was based on a literature review and a discussion in a focus group with representatives of the general population and was refined in expert interviews. Each alternative represented a combination of fictitious drugs and pathology.

The study was conducted among the general population in two steps: pilot study and main study. The pilot study represented a preliminary phase undertaken to test the questionnaire and to collect prior information on attributes weights. This prior information was used to improve the statistical efficiency of the main study. The pilot study enrolled 30 participants and was followed by a series of cognitive debriefings to assure good understanding of the questionnaire and adjust the wording.

The following sections describe all the study phases in detail.

### Attributes and Their Levels

The basis for the selection of attributes was a targeted literature review on elements of ODs value (12). The developed search strategy did not target price determinants or elements of ODs value specifically but focused on any matter related to HTA approaches and methodology, as well as on the social value of ODs. The search strategy was developed using relevant keywords and Medical Subject Headings (MESH) terms (see Supplementary Material). The search was conducted using the Medline bibliographical database and was run via the Ovid interface. The search was completed by a gray literature search. HTA agencies' and regulatory bodies' official websites were screened non-systematically to identify any publication of interest.

No specific restriction based on geographical scope or publication data was applied. Only publications in English were retrieved. The Medline search using the developed strategy resulted in 718 relevant references and the final selection after the screening included 29 references. The selected publications were analyzed and all cited elements of the ODs value were extracted to form a preliminary list of attributes.

The most important attributes were then selected in a focus group conducted with representatives of the French general population. Five participants were enrolled. A semi-directive discussion guide was developed and used to structure the discussion.

During the focus group, the participants were presented with basic information on the principles of healthcare resource allocation, the importance of HTA and economic evaluation, as well as specificities of drugs for rare diseases. The importance of each criterion from the preliminary list was discussed with an objective to select the most relevant attributes.

Potential attributes were not limited by the preliminary list, participants were free to propose any other characteristics they found relevant. Attribute levels were fixed after the selection of attributes was finished. The discussion was continued until achieving a consensus among all participants. The list of attributes after this step included: disease severity, unmet needs, disease prevalence, expected impact of the drug on disease mortality and morbidity, drug safety, certainty in the expected impact, as well as drug cost per patient.

In the next step, the developed list of attributes and their levels was discussed and validated with two experts: one expert specializing in economic evaluation and one expert specializing in rare diseases. A 1-h telephone interview was carried out with each expert. The disease severity attribute was split in two: disease mortality and morbidity, following the experts' suggestion. The attribute levels were defined in such a way that the range would cover most of the treatments approved or considered for approval in France at the time of the study. For an annual cost of treatment, the range also takes into consideration findings from the focus group discussion on willingness to pay.

Finally, the attributes and their levels were validated in the pilot study and cognitive debriefings.

The final list of attributes and their levels is presented in Table 1.

**Table 1:** List of attributes and their levels included in the main study.

**Disease-associated mortality**
- The disease does not decrease life expectancy
- With no treatment, the patient will die at the age of 40 years
- With no treatment, the patient will die at the age of 20 years
**Disease-associated disability**
- Patient does not face difficulties in his everyday life, but some activities requiring a lot of effort may be contraindicated (e.g., sport)
- Patent may face difficulties in his everyday life, but remains independent
- Patient needs assistance constantly
**Unmet needs**
- No other treatment exists
- Other treatments are available, but their performance is limited
- Other treatments are available, and their performance is high
**Number of patients in France**
- 500 patients
- 2,000 patients
- 10,000 patients
- 20,000 patients
**Expected impact on disability**
- With Treatment 1(2) the disease disability is totally eliminated
- With Treatment 1(2) the patient does not face difficulties in his everyday life, but some activities requiring a lot of effort may be contraindicated (e.g., sport)
- With Treatment 1(2) the patient may face difficulties in his everyday life but remains independent
**Expected impact on mortality**
- Treatment 1(2) increases life expectancy by 30 year
- Treatment 1(2) increases life expectancy by 10 years
- Treatment 1(2) increases life expectancy by 2 years
- Treatment 1(2) does not impact life expectancy
**Drug safety**
- Treatment 1(2) can cause adverse effects leading to hospitalization or permanent disability
- Treatment 1(2) may cause adverse effects with moderate impact on health which will disappear when the patient stops the treatment
- Treatment 1(2) does not cause adverse effects
**Certainty regarding available evidence**
- We have great confidence in the expected benefit of the drug
- We have moderate confidence in the expected benefit of the drug
- We have little confidence in the expected benefit of the drug
**Additional annual cost per patient**
- €10,000 per year
- €50,000 per year
- €200,000 per year
- €500,000 per year

### Experimental Design

The experimental design defines combinations of attributes levels for all choice alternatives presented to participants. This study used a D-efficient design. Efficient designs minimize the variance around estimates and allow using prior information to increase the statistical efficiency of the design (10). Both pilot and main studies were designed using a D-efficient approach. The pilot study design was generated using zero priors, while the main study used the results of the pilot study as prior values.

**Figure 1:**
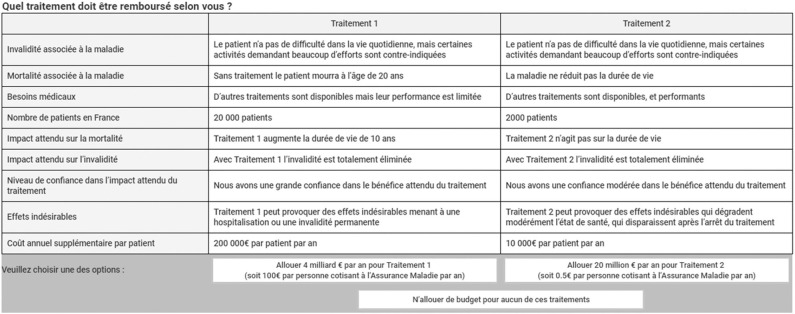
Example of the choice scenario presented to participants.

In each choice task, members of the general public were asked to choose which of the two treatments (called Treatment 1 and Treatment 2) targeting different diseases should be reimbursed in France. Since the choice of either of them should not be associated with any utility by itself (regardless of the associated attribute levels), the design was unlabeled. The choice alternatives were not meant to represent any known disease in particular. Respondents were also given the possibility to approve none of the two presented treatments. Figure 1 provides an example of a choice scenario as presented to respondents.

The number of choice situations was limited to 36 and they were grouped into 3 blocks of 12 questions. Each participant responded to one block of questions. To facilitate the exercise, the number of attributes which could take different levels between two alternatives in each choice situation was limited to seven (i.e., at least two attributes took the same level for both alternatives in each choice situation). This condition was imposed by introducing constraints in the experimental design. Other constraints were also introduced to the design to exclude some implausible combinations of attributes.

Ngene software version 1.1.2 was used to generate the experimental design.

### Participants and Survey Administration

The study intended recruiting a large sample of French general population representatives. Although there exists no consensus on calculations of a sample size to allow for a robust and precise estimation of attribute weights, simulations demonstrated a rapid increase in the precision at sample sizes <150 and, then, flattening out at around 300 observations (13). According to the ISPOR Task Force Report on DCE construction, different research questions may have different requirements on the sample size, and measurement errors may significantly impact the precision of estimates (10). For the current study, a sample size of 1,000 participants was considered to be sufficient, which resulted in more than 300 participants per questionnaire.

Participants were recruited by an external subcontracting party which was responsible for distributing the survey. Quotas on age, gender, region, and working status were applied to ensure the representativeness of the sample. The survey was designed as a web-based questionnaire. Participants were allocated to one of the three blocks based on the least filled criteria.

The survey started with an introduction which presented the context of the study and explained the task. The introduction provided a general and brief description of rare diseases, without referring to any specific disease. The participants were told that because of limited healthcare budgets, all developed drugs cannot be covered by the insurance and health authorities have to decide which drug should be reimbursed. Disease characteristics (i.e., disease severity and natural history), drug's efficacy and safety profile demonstrated in clinical trials, as well as economic considerations (i.e., cost of treatment), were cited as the primary criteria for the decision. The participants were also informed about the specificities of rare diseases: small number of patients, high severity, and the lack of alternative treatments. The reasons for high prices of ODs were explained.

Thereafter, the participants were asked to imagine themselves in the position of healthcare authorities and make a choice between two alternative treatments which were described according to the selected attributes. In order to sensitize respondents to treatment costs, they were told that choosing a treatment would require allocating an amount of money necessary to treat all patients suffering from the disease during a 1-year period. This sum was assumed to remain constant over time. Moreover, it was specified that allocating resources to a treatment would mean that they could not be spent on other diseases or an increase in insurance premium would be needed. To facilitate the choice task, the total expenses for each drug, as well as the resulting potential increase in premium, were presented for each drug. Buttons for choosing drugs were provided with the following legend: “Allocate € XXX per year for Treatment 1/2 (€XXX per year per person contributing to the national insurance plan).”

### Statistical Methods

Demographic characteristics of the participants were studied using descriptive statistics. A mixed logit model with random effects was constructed to derive the estimates of attributes' weights. The model included all attributes as well as a dummy variable corresponding to the opt-out alternative. Additionally, an interaction between the number of patients and per patient treatment cost, representing the budget impact of the treatment, was added in the model.

All attributes were initially considered as categorical variables. The number of patients and per patient treatment cost attributes, as well as their interaction could be transformed to continuous variables based on the following conditions: the linearity of their effects had to be confirmed by visual observation and minimization of Akaike information criterion. The threshold for statistical significance was set at 0.05. All analyses were conducted in R version 3.3.2.

Subgroup analyses were performed on the following participant subgroups: people who claimed to be aware of rare disease vs. those not aware of rare diseases, and people who knew someone suffering from a rare disease vs. those who did not.

## Results

A total of 958 persons participated in the main survey. Women represented 51% (*n* = 491) of the participants. The age of all respondents varied between 18 and 89 years old with the mean age of 47.5 years. The sample was considered being representative of the French general population regarding sociodemographic characteristics (see Table 2). The majority (*n* = 840, 88%) of the participants claimed to be aware of rare diseases. Moreover, a total of 169 respondents declared that someone from their entourage or themselves suffered from a rare disease.

**Table 2:** Sociodemographic characteristics of study population.

	**Number of persons (%) (*n* = 958)**
**Gender**	
Female	491 (51.3%)
Male	467 (48.7%)
**Age**	
Mean (SD)	47.5 (16.5)
18–24	100 (10.4%)
25–34	156 (16.3%)
35–44	169 (17.6%)
45–54	173 (18.1%)
55–64	132 (13.8%)
65+	228 (23.8%)
**Family status**	
Single	264 (27.6%)
Married	461 (48.1%)
Divorced	96 (10.0%)
Civil partnership	61 (6.4%)
Other	47 (4.9%)

The rates of satisfaction and interest in the survey were high: 46 and 39% of the participants claimed being “Extremely satisfied” or “Very satisfied” with the survey, and 45 and 39% of the participants found the survey “Extremely interesting” or “Very interesting.”

The results of the mixed logit model are presented in Table 3. The initial model included all attributes, as well as a dummy variable corresponding to the opt-out. Subsequently, an interaction between the number of patients and per patient treatment cost was added in the model and was statistically significant. The linearity of the effect analyzed by visual observation was confirmed for the interaction, but not for the main effects of the number of patients and treatment cost attributes. The interaction was thus transformed as a continuous variable, which also improved the AIC.

**Table 3:** Attribute weight estimates: results from the main study.

**Attribute**	**Estimate**	**SE**	***P*-value**
**Disease mortality**			
No reduction of life expectancy (ref)	-	-	-
Life expectancy of 40 years	0.051264	0.09068	0.571851
Life expectancy of 20 years	0.29026	0.098836	0.003316
**Disease disability**			
Mild disability (ref)	-	-	-
Moderate disability	−0.00903	0.063749	0.887368
Severe disability	−0.10694	0.061096	0.080057
**Unmet needs**			
Effective alternative treatments (ref)	-	-	-
Alternative treatments with limited effectiveness	0.29125	0.067112	<0.0001
No alternative treatments	0.17761	0.054199	0.001049
**Number of patients**			
500 (ref)			
2,000	−0.15033	0.077468	0.052313
10,000	0.29309	0.064857	<0.0001
20,000	0.23332	0.081416	0.004159
**Expected impact on mortality (life expectancy level with treatment)**			
Increase in life expectancy of 30 years	-	-	-
Increase in life expectancy of 10 years	−0.96417	0.074194	<0.0001
Increase in life expectancy of 2 years	−0.91935	0.071289	<0.0001
No impact on life expectancy	−1.1118	0.071977	<0.0001
**Expected impact on disability (disability level with treatment)**			
No disability (ref)	-	-	-
Mild disability	−0.18885	0.059165	0.001414
Moderate disability	−0.26117	0.061169	<0.0001
**Drug safety**			
No adverse events (ref)	-	-	-
Mild adverse events	0.033187	0.075388	0.659775
Severe adverse events	−0.46209	0.053652	<0.0001
**Certainty regarding available evidence**			
Great confidence (ref)	-	-	-
Moderate confidence	−0.55968	0.066573	<0.0001
Little confidence	−0.83779	0.06959	<0.0001
**Annual cost per patient**			
€10,000 (ref)	-	-	-
€50,000	0.21602	0.069432	0.001863
€200,000	−0.0878	0.068821	0.202024
€500,000	−0.23455	0.11722	0.045404
**Opt-out**	−4.4471	0.16348	<0.0001
**Interaction of number of patients and treatment cost**	−8.9E-11	2.13E-11	<0.0001

*SE, standard error*.

The values in Table 3 are the estimates of the mixed logit model coefficient. Positive values indicate that the corresponding level was preferred to the reference level and higher values indicate a higher degree of preference.

The highest weight estimates were observed for drug impact on disease mortality. The respondents were more likely to choose treatments that increase life expectancy by 30 years rather than treatments that extend patient life by 10 years (−0.964, *p* < 0.0001), 2 years (−0.919, *p* < 0.0001) or treatments that do not have an impact on life expectancy (−1.112, *p* < 0.0001).

The second highest impact on the respondents' choices was associated with the uncertainty regarding drug effect. The participants valued drugs with a great confidence regarding their therapeutic effect compared to treatments associated with moderate confidence (−0.560, *p* < 0.0001) or little confidence (−0. 838, *p* < 0.0001).

Additionally, the respondents were sensitive to drug safety and availability of alternative therapies. The respondents were more likely to choose treatments without available alternative options (0.178, *p* = 0.0010) or when the effectiveness of alternative options was limited (0. 291, *p* = 0.0003) compared to drugs with effective alternative available. Drugs with severe adverse events were less likely to be chosen compared to drugs that do not cause adverse events (−0.462, *p* < 0.0001). The respondents were more tolerant regarding mild adverse events. No statistically significant difference in preferences was observed between drugs causing mild adverse events and drugs without adverse events.

The number of patients and per patient treatment costs also demonstrated a significant impact on the participant's choices. Respondents were willing to treat more patients at a given total cost. Statistically significant results were observed for the comparison between 500 and 10,000 patients (0.293, *p* < 0.0001) and 20,000 (0.233, *p* = 0.0041). Regarding treatment costs, lower preferences for higher costs were observed only for the comparison of €500,000 per patient vs. €10,000 per patient (−0.235, *p* = 0.04540), while €50,000 per patient was preferred to €10,000 per patient (0.216, *p* = 0.0019). Drugs with a lower budget impact were preferred (*p* < 0.0001), but the effect of budget impact was not large enough to outweigh the effect of patient number unless the cost per patient was very high. Thus, if we consider a treatment with an annual cost of €200,000, the difference in utility between scenarios with 10,000 and 500 patients treated is positive (0.29309 − 8.9 × 10^−11^ × (10000 − 500) × 200000 = 0.124), indicating that people would be in favor of treating 10,000 patients rather than 500. However, for a treatment with an annual cost of €500,000, people would prefer if 500 patients were treated rather 10,000 (difference in utility for 10,000 treated patients vs. 500: −0.130).

Disease characteristics (disability and mortality) had only a very moderate impact on participant choices. Although there existed a trend toward higher preferences for more disabling diseases, neither moderate nor severe initial disability reached the threshold for statistical significance in the comparison vs. mild disease disability. For disease mortality, significantly higher preferences were observed only for diseases with the shortest life expectancy (20 years) compared to diseases that did not shorten patient life (0.290, *p* = 0.0033).

Estimates of the willingness-to-pay for a life-year (LY) gained can be derived from the coefficient estimates for the number of patients, impact on mortality and budget impact (interaction of annual cost per patient and the number of patients). They range from €21,401 per LY gained when considering a treatment adding 10 years of life for 20,000 patients or €52,618 when considering a treatment adding 30 years of life for 10,000 patients.

Results were generally similar between respondents who claimed to be aware of rare diseases and those who did not, as well as those who knew a patient with a rare disease and those who did not. Among notable differences, the trend for higher preferences toward more prevalent diseases was not observed in the subgroup of participants who were not aware of rare diseases. The participants who had rare disease patients in their entourage demonstrated higher acceptance for drugs with higher per-patient costs.

## Discussion

Identifying elements of an ODs value and their relative importance to the society is an important step in making recommendations regarding public funding of medicines for rare conditions. The current study aimed at answering two main questions: what are the characteristics of ODs that define an ODs value according to the society and what are the relative weights that the society places on these characteristics?

Based on the literature review and qualitative research, we found that the evaluation of ODs should consider nine key attributes representing disease severity and prevalence, drug efficacy and safety profile, uncertainty around its therapeutic effect and per patient cost. The DCE showed that all these attributes mattered significantly from the perspective of members of the general public, except for disease-associated disability, and the attributes with the largest influence were the impact of treatment on mortality and the degree of confidence in clinical evidence.

Respondents attached substantial value to treatments with a dramatic increase in life expectancy (30 years), which may have been perceived as approaching a cure. They also attached value to treatments with an incremental gain in life-expectancy (2–10 years), compared to treatments with no impact on life expectancy, but that value was much lower than for a gain of 30 years and was quite similar between gains of 2 and 10 years. This result suggests that the value placed by people for a dramatic gain in life expectancy exceeds the sum of values attached to incremental gains adding up to the same number of life-years. This contradicts the findings from Hampson et al., who found that people did not place value on a treatment being a cure *per se* (14).

The impact of treatment on the disability was considerably less valued than the impact on mortality. Thus, the respondents privileged extending life expectancy rather than improving patient quality of life. This may be related to the fact that this study reflects social preferences (i.e., preferences for others) rather than individual preferences (i.e., preferences of people for themselves). It has been previously noted that valuations of life based on social preferences place more emphasis on duration of life, and less on quality of life, when compared to valuations based on individual preferences (15).

Respondents placed little value on disease-related attributes, i.e., disease-related disability and mortality. Some previous studies demonstrated that when analyzing a patient or public perspective, higher preferences were placed on the impact of the disease and unmet needs rather than the drug impact (1, 9, 16, 17). Fueled by the idea of fairness in the allocation of healthcare resources, respondents were willing to treat the neediest patients regardless of their ability to benefit from a treatment or associated costs (18). These findings were not confirmed in the present study where greater weights were associated with therapeutic benefit of the drug.

The second most valued attribute was the certainty regarding drug therapeutic effect. The participants were highly sensitive to the lack of confidence in expected therapeutic benefit. While authorities generally demonstrate high acceptance of uncertainty for ODs, whether on regulatory or HTA level (19), the society seems to be less willing to allocate budgets for treatments with an uncertain therapeutic effect.

As expected, the budget impact had a negative influence on the magnitude of preference for a treatment. It is noteworthy that the estimated costs per life-year gained are more or less in line with those accepted for non-orphan drugs, and relatively low when compared to annual costs of recently approved orphan drugs (e.g., elosulfase alfa, carglumic acid, velaglucerase). Interestingly, people were not indifferent to the drug price for a given level of budget impact. An annual cost of €50,000 per patient was preferred to €10,000. This finding, which appears irrational, may be associated with a perception among respondents that “cheap” drugs have less value. There was no significant preference between treatments with costs of €10,000 and €200,000, however a treatment with an annual cost of €500,000 was considered as too expensive.

The relationship between the level of preference and the number of patients was not monotonous. This may have resulted from two different ways of thinking when considering this attribute: people are likely to prefer treatment strategies benefitting a greater number of people, but they also want to support options for patients with very rare diseases, sometimes at the expense of patients suffering from more prevalent diseases.

The current study was the first attempt to identify preferences of the French general population for OD funding. The study enrolled a large number of participants and employed a robust methodology. The sample of participants was representative of the French adult population in terms of age and gender. However, the lack of awareness regarding rare diseases and drug funding in general might have led to an incomprehension and inability to fully understand the consequences of the choices.

Another study limitation is that several elements of drug social value that might be lost from consideration in the present study. Particularly, Richardson et al. demonstrated in their survey a social will to abandon the strategy of maximizing health and even to sacrifice health gains in exchange for sharing (20). The notion of sharing, very close to the notion of equal rights for treatment, was found to be the main driver in the allocation of the resource. However, integrating the notion of sharing, where each choice situation should be treated independently, was not feasible in this DCE.

To conclude, the determinants of social preference for orphan drugs are complex. This study suggests that people value the impact of drugs on the duration of life, and in particular large gains in life expectancy, much more than quality of life. It also shows major concern for the quality of evidence. Although the study suggests some concern for equity, estimates of the willingness-to-pay for orphan drugs are not higher than those usually cited for other drugs. Further research is needed to clarify how equity is valued by society.

## Data Availability Statement

The datasets for this article are owned by Creativ-Ceutical and are not publicly available. Requests to access the datasets should be directed to creativ@creativ-ceutical.com.

## Ethics Statement

Ethics approval for this study was not required in accordance with national legislation and institutional requirements.

## Author Contributions

MT: study conception. AM, JD, KT-D, OC, and SA: literature review and data collection. AM, KT-D, and SA: drafting of the manuscript. All authors: contributed to the study design, interpretation of data, critical revision of the intellectual content and final approval of the version to be published, and agree to be accountable for all aspects of the work.

## Conflict of Interest

The authors declare that this study received funding from Creativ-Ceutical, which is the employer of the authors. Therefore the funder was involved in all aspects of the study.
