# Supplementary material for: Social Preferences for Orphan Drugs: A Discrete Choice Experiment Among the French General Population
Source: Front Med (Lausanne). 2020 Jul 17;7:323. doi: 10.3389/fmed.2020.00323 (PMC7379418; doi:10.3389/fmed.2020.00323)
Supplement: Supplementary file 1 [file Table_1.docx]

**Literature search strategy**

| 1 | Rare disease/Orphan drugs | exp Orphan Drug Production/ | 872 |
| --- | --- | --- | --- |
| 2 |  | exp Rare Diseases/ | 6550 |
| 3 |  | (rare disease? or orphan disease? or rare disorder? or orphan disorder? or rare condition? or orphan drug? or orphan product? or ultrarare disease? or highly specialized technolog* or orphan medicinal product? or ultraorphan drug? or ultraorphan disease? or neglected disease?).ti,ab. | 30235 |
| 4 | HTA | exp Economics, Pharmaceutical/ or exp Economics, Medical/ or exp Economics/ | 520580 |
| 5 |  | exp Decision Making/de, es [Drug Effects, Ethics] | 13567 |
| 6 |  | exp Technology Assessment, Biomedical/ | 9704 |
| 7 |  | exp Insurance, Health, Reimbursement/ or exp Reimbursement Mechanisms/ or exp Reimbursement, Incentive/ | 40602 |
| 8 |  | exp Evaluation Studies as Topic/ec [Economics] | 1543 |
| 9 |  | exp "Costs and Cost Analysis"/ | 194785 |
| 10 |  | exp Drug Costs/ | 13023 |
| 11 |  | exp Health Policy/ec [Economics] | 8429 |
| 12 |  | exp Health Resources/ec, es [Economics, Ethics] | 2216 |
| 13 |  | exp Social Values/ | 19063 |
| 14 |  | exp Quality-Adjusted Life Years/ | 7901 |
| 15 |  | exp Cost-Benefit Analysis/ | 64802 |
| 16 |  | (multicriteria decision analys#s or multi-criteria decision analys#s or multi criteria decision analys#s or mcda).mp. | 285 |
| 17 |  | (economic evaluation or technology assessment).ti,ab. | 8523 |
| 18 |  | (pricing or funding or reimbursement).ti,ab. | 46917 |
| 19 | Hits of Rare disease/Orphan drugs | 1 or 2 or 3 | 35582 |
| 20 | Hits of HTA | 4 or 5 or 6 or 7 or 8 or 9 or 10 or 11 or 12 or 13 or 14 or 15 or 16 or 17 or 18 | 583982 |
| 21 | Hits of Rare disease/Orphan drugs and HTA | 19 and 20 | 820 |
| 22 |  | limit 21 to english language | 718 |
